# The Role of Salivary Diagnostics in Early Detection of Systemic and Oral Diseases: A Comprehensive Review

**DOI:** 10.7759/cureus.100313

**Published:** 2025-12-29

**Authors:** Shalin D Shah, Sudarshan Gupta, Pramod Kumar Pamu, Jay Priteshkumar Shukla, Subash Nayak, Kumarjyoti Chatterjee

**Affiliations:** 1 Oral and Maxillofacial Surgery, Government Dental College and Hospital, Ahmedabad, IND; 2 Department of Pathology, Virendra Kumar Sakhlecha Government Medical College, Neemuch, IND; 3 Pathology and Laboratory Medicine, Nizam's Institute of Medical Sciences (NIMS), Hyderabad, IND; 4 Anesthesiology, Dr. N D Desai Faculty of Medical Science and Research, Dharmsinh Desai University, Nadiad, IND; 5 Orthodontics and Dentofacial Orthopaedics, Utkal University, Bhubaneswar, IND; 6 Oral Pathology, Awadh Dental College and Hospital, Jamshedpur, IND

**Keywords:** biomarkers, early detection, point-of-care devices, precision health, salivary diagnostics, salivary proteomics

## Abstract

Early diagnosis of oral and systemic diseases remains a major challenge due to limitations in conventional invasive procedures. This review addresses the critical need for non-invasive, accessible diagnostics by examining the potential of saliva as an alternative diagnostic fluid. The main objective is to synthesize current knowledge on salivary biomarkers and technological innovations that enhance early detection capabilities. Methodologically, the review integrates evidence from proteomic, genomic, and metabolomic studies, as well as recent advances in microfluidic point-of-care devices. Key findings highlight that saliva contains a rich array of biomarkers, including cytokines, microRNAs, and extracellular vesicles, that can accurately reflect both local oral pathologies, such as dental caries and oral cancer, and systemic conditions, including diabetes, cardiovascular diseases, autoimmune disorders, and neurodegenerative diseases. The analysis further emphasizes that portable biosensors and lab-on-chip platforms are rapidly improving diagnostic sensitivity and enabling decentralized testing. Notwithstanding these encouraging advancements, issues with biological variability, standardization, and regulatory validation still exist. This review underscores the significance of interdisciplinary collaboration to overcome these barriers and fully integrate salivary diagnostics into routine healthcare. The implications are profound: widespread adoption could transform preventive medicine by providing patient-friendly, real-time monitoring tools that improve health outcomes globally.

## Introduction and background

One of the most crucial tasks in the modern healthcare system is the early diagnosis of the disease. Early diagnosis is also very important as it could result in better prognosis, lower cost of treatment, and better quality of life for the patients due to early intervention and management [1]. Blood sampling, tissue biopsy, and imagiological examinations, including radiographic techniques such as sialography, panoramic radiography, and cone-beam computed tomography (CBCT), as well as non-radiographic magnetic resonance imaging (MRI), are traditional diagnostic approaches used for decades [2]. The standard methods, however, are usually invasive, time-consuming, and sometimes inconvenient in frequent observation or mass screening exercises [3]. Specifically, the pediatric, geriatric, or medically compromised populations are particularly vulnerable to the added discomfort, risk of the procedures, and compliance when undergoing conventional diagnostic procedures [3,4]. This review specifically synthesizes evidence across key salivary analyte classes, including enzymes (e.g., amylase, lysozyme), inflammatory cytokines, microRNAs, and extracellular vesicles, alongside metabolomic profiles that reflect oral and systemic pathophysiology. We also outline methodological considerations (collection, processing, standardization) and technological advances (point-of-care platforms, biosensors, and salivaomics) to provide a coherent flow from biological foundations to clinical translation [1,4,5].

In that regard, the creation of patient-friendly, accessible, and non-invasive diagnostic instruments has become a priority in the field of translational research and clinical innovation [5]. The development of point-of-care technologies and biomarker discovery has opened the door to the fact that alternative biofluids, such as urine, tears, serum, cerebrospinal fluid, and sweat, and molecular targets can be used to detect the disease. Due to its ease of collection and the abundance of information it may provide about oral and systemic health, saliva is one of these items that has attracted a lot of scientific and clinical attention [6,7]. Because it contains secretions from both the major and minor salivary glands, gingival crevicular fluid, mucosal transudate, serum components, bacteria, epithelial cells, and food debris, saliva is a complex biological fluid [1,8]. Such heterogeneity implies that saliva has a wide repertoire of molecules, such as electrolytes, hormones, immunoglobulins, enzymes, cytokines, nucleic acids, and microbial signatures, many of which reflect the physiological and pathological conditions of the body [9,10]. The saliva has one of the unique properties because it can represent local oral conditions and systemic changes; thus, it is a potential source of integrative diagnostics. Biomarkers (salivary analytes) of infectious diseases, malignancies, autoimmune disorders, metabolic syndromes, and neurological diseases can be found [2,6,11]. Cytokines and other inflammatory mediators are found in the saliva of patients with oral squamous cell carcinoma, periodontal disease, and systemic infections [3,12]. Prominent examples include interleukins such as IL-1β, IL-6, and IL-8; tumor necrosis factor-alpha (TNF-α); interferon-gamma (IFN-γ); and chemokines like CXCL8 and CCL2, which serve as key salivary biomarkers reflecting inflammatory and oncogenic activity. Similarly, salivary proteomic and metabolomic signatures have also been shown to have the potential to distinguish between malignant and benign lesions, as well as predict disease progression [4,8,13].

Salivary testing has some unique benefits compared to serum or tissue diagnostics. Collection of the samples is non-invasive, generally less stressful for most patients, though not entirely stress-free, as subjective factors can influence patient comfort. For example, certain procedures, such as salivary flow rate examinations, may cause mild discomfort or anxiety for some individuals, thus amplifying patient acceptability and making it possible to obtain repeated samples over time [14]. This may be especially useful to longitudinally follow the disease biomarkers or to screen at-risk populations who are unwilling to undergo more invasive tests. Also, there is no need for trained phlebotomists and blood handling biohazard risks since saliva is collected [5,15]. Salivary diagnostics are also amenable to rapid point-of-care applications. Recent innovations in technologies, including lab-on-a-chip systems, biosensors, microfluidics, etc., have helped design portable platforms that can identify low-abundance salivary biomarkers with high sensitivity and specificity [7,16]. The innovations are leading to a paradigm shift to personalized, decentralized, and prevention-based models of healthcare.

This review aims to summarize the current state of salivary diagnostics and its role in the early detection of systemic and oral diseases. It covers physiological and methodological aspects, collection procedures, analytical methods, and biomarker validation crucial for test accuracy. The review discusses salivary biomarkers in oral diseases like caries, periodontitis, and oral cancer, as well as systemic conditions such as diabetes, cardiovascular, autoimmune, infectious, and neurodegenerative diseases. It highlights advances in salivary proteomics, metabolomics, and salivaomics in precision medicine. The review also identifies challenges like biological variability, lack of standardization, and regulatory issues, and suggests solutions and future directions. Overall, it emphasizes the potential of salivary diagnostics to transform clinical practice through early, convenient disease detection.

Methodology

A narrative search was conducted in PubMed/MEDLINE, Scopus, and Web of Science (last search: [insert Month Year]). Search strings combined the terms saliva/salivary with diagnostics, biomarkers, microRNA, proteomics, metabolomics, point-of-care, and disease descriptors (oral cancer, periodontitis, diabetes, cardiovascular, autoimmune, infectious, neurodegenerative). Peer-reviewed, English-language human studies (original research, systematic/narrative reviews, and methodological papers) were included; conference abstracts and non-peer-reviewed sources were excluded. Titles and abstracts were screened for relevance with full-text verification. As this is a narrative synthesis, no formal risk-of-bias instrument was applied; potential selection bias is acknowledged and mitigated through multi-database searching, explicit inclusion/exclusion criteria, and preference for recent, higher-quality evidence.

## Review

Physiology and composition of saliva

Major and Minor Salivary Glands

The salivary glands that create saliva include the parotid, submandibular, and sublingual glands, as well as numerous smaller salivary glands dispersed throughout the oral mucosa. The parotid glands, which are in front of the ears, produce a serous, watery secretion that is high in amylase and other digestive enzymes. The submandibular glands, which are beneath the floor of the mouth, secrete a mixed seromucous fluid that makes a large contribution to resting salivary flow [10,11]. Sublingual glands are glands found beneath the tongue, and they produce mainly mucous secretions, which lubricate the oral cavity [12]. The minor salivary glands are located throughout the lips, cheeks, palate, and tongue and secrete mucins and antimicrobial peptides vital to mucosal protection and innate immunity [13].

The autonomic nervous system and circadian rhythms affect the composition of each gland, with their protein and electrolyte composition being different [14]. The parasympathetic stimulation tends to cause a profuse, watery secretion, whereas the sympathetic stimulation produces less volume with organic constituent enrichment [15]. Combined secretion of all these sources produces whole saliva, which is a dynamic, multifactorial biofluid that reflects oral and systemic physiology [16].

Constituents of Saliva

Saliva consists of water (about 99%) and various organic and inorganic solutes (about 1 percent) [17]. In order to maintain mineral homeostasis, pH buffering, osmotic balance, and microbial control, sodium, potassium, chloride, bicarbonate, calcium, and phosphate are necessary electrolytes [18].

Proteins and enzymes form another essential component. The most abundant salivary proteins are amylase, which begins the digestion of starch and controls bacterial adhesion [11,19]. It is noteworthy that the composition of saliva differs between major and minor salivary glands. The major glands: parotid, submandibular, and sublingual; primarily secrete serous fluids rich in enzymes such as amylase, lysozyme, and peroxidase, contributing to digestion and antimicrobial defense. In contrast, the numerous minor salivary glands distributed throughout the oral mucosa produce predominantly mucous secretions composed of mucins, glycoproteins, and immunoglobulins, which play vital roles in lubrication, mucosal protection, and maintenance of oral homeostasis.. Mucins (e.g., MUC5B and MUC7) are reserve substances of high molecular weight and are glycoproteins that lubricate the mucous layer and trap the pathogens [20]. The adaptive immune protection is achieved by secretory immunoglobulin A (sIgA), which neutralizes microbial antigens, whereas lactoferrin, lysozyme, and histatins have innate antimicrobial properties [21].

Hormones, growth factors, and cytokines involved in tissue repair and inflammatory signaling are also found in saliva [22]. Extracellular DNA, microRNAs, and messenger RNAs have also been identified as nucleic acids that are biomarkers of local diseases such as oral squamous cell carcinoma, periodontitis, and dental caries, and systemic pathology [23]. As an example, microRNA signatures have been observed in oral cancers and cardiovascular disease patients, providing five insights into the gene expression patterns with regard to the disease progression [24]. Salivary metabolomics also emphasizes low-molecular-weight molecules, including organic acids, amino acids, and short-chain fatty acids (SCFAs) that can be altered with metabolic or inflammatory disorders [25].

All of these components create a rich biochemical environment that makes saliva a very useful diagnostic fluid that can be utilized to identify a vast number of pathological events [12,17,20].

Saliva as a Reflective Fluid

The process of the appearance of systemic biomarkers in saliva has several pathways. Initially, small molecules and specific hormones diffuse through the acinar or ductal epithelial cells using passive diffusion into the glandular lumen [18]. Second, active transport processes are able to secrete certain proteins or electrolytes selectively [26]. Third, the serum contents can enter the saliva via the tight junctions between acinar cells through ultrafiltration [19]. Moreover, cytokines, immunoglobulins, and microbial by-products are added to the whole saliva by the gingival crevicular fluid, whose origin is serum exudate and local inflammatory reactions [13,21]. Such a combination of transcellular, paracellular, and exogenous pathways makes saliva a reflection of local and systemic physiology.

**Figure 1:**
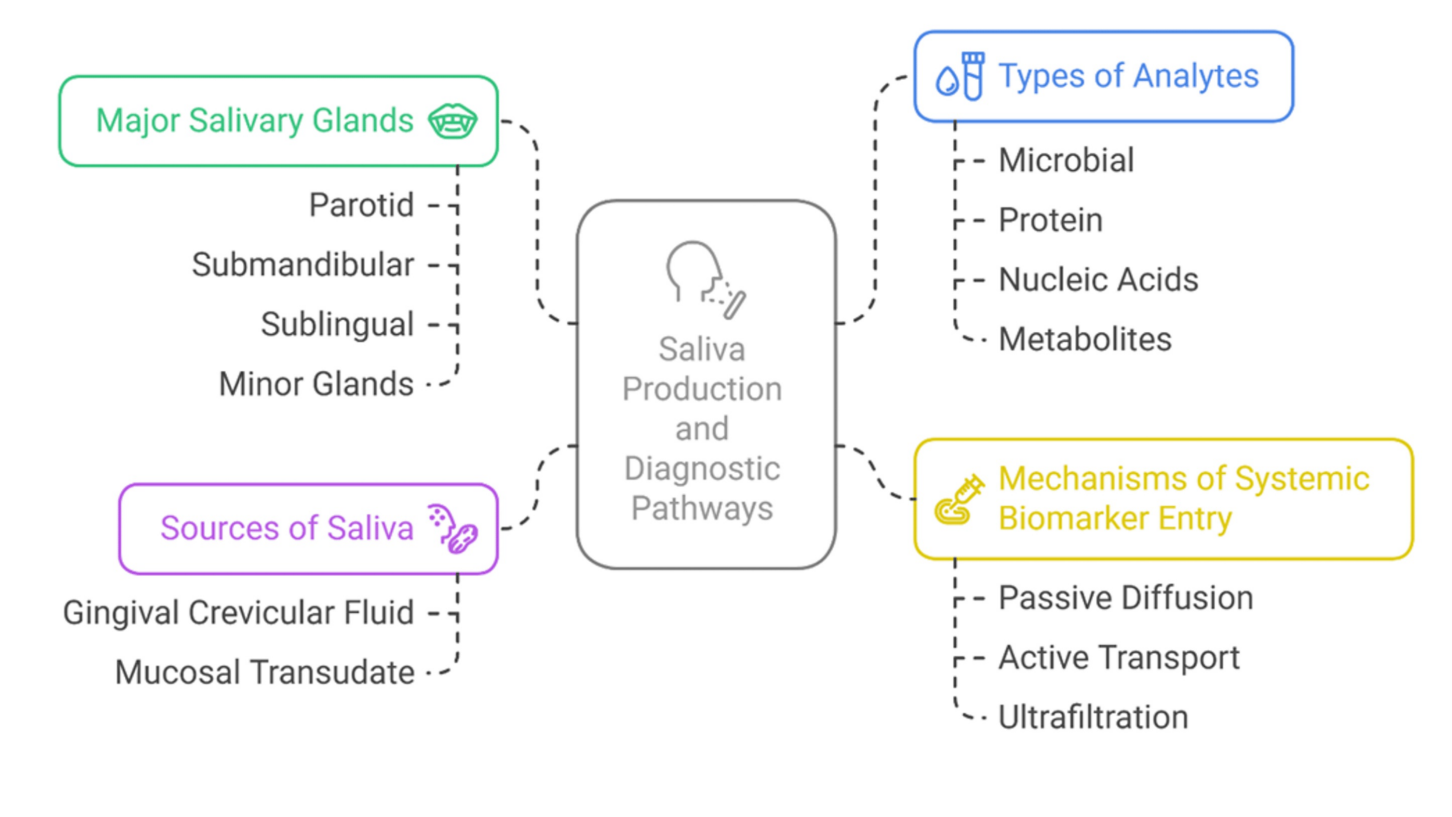
Anatomical Sources of Saliva and Biomarker Entry Pathways Created by authors

Evidence is also emerging that salivary extracellular vesicles (EVs) can be used to deliver proteins, nucleic acids, and lipids to the oral cavity, which are released by distal tissues [23,27]. These vesicles can be used as stable biomarker carriers, as labile molecules are not degraded by enzymes, and salivary diagnostics will be more reliable. As an illustration, recent research has shown that salivary exosomal microRNAs may distinguish between healthy people and patients with oral squamous cell carcinoma or systemic disorders, including Sjögren syndrome and Alzheimer's disease [23,24,28]. Figure 1 shows the major and minor salivary glands, pathways of systemic biomarker entry into saliva, and the origin of diagnostic analytes.

Methodological considerations in salivary diagnostics

Saliva Collection Methods

Proper collection is foundational to accurate salivary diagnostics. Saliva can be gathered in stimulated or unstimulated states. Unstimulated whole saliva will be indicative of basal secretion and is normally obtained through passive drooling or spitting into a sterile container without any external stimulation [14,16]. This is the preferred method where biomarkers under investigation are dependent on resting glandular activity, e.g., baseline levels of cytokines or hormones [15]. In contrast, stimulated saliva is provoked by gustatory agents (e.g., citric acid), mechanical chewing (paraffin wax), or olfactory stimuli [11]. Stimulation enhances the flow rates of saliva and can dilute some analytes, but also helps to collect in people with hyposalivation or xerostomia [12]. Researchers should take into account that stimulation changes electrolyte composition, pH, and protein levels that may confound the interpretation of results [13].

In addition to whole saliva, gland-specific collection provides specific analysis of contributions by individual glands. As an example, parotid saliva may be collected with the help of Lashley cups attached to the Stensen duct, and submandibular/sublingual secretions may be isolated with special cannulation equipment [10,19]. However, it should be noted that these gland-specific collection methods can be challenging in pediatric and special needs patients due to limited cooperation, anatomical variations, and discomfort during the procedure, which may restrict their routine clinical applicability in such populations. Sampling at a gland level gives a more uniform analyte concentration and allows pathology (e.g., salivary gland tumor or localized inflammation) to be localized [17]. Pre-collection procedures (fasting, oral hygiene, avoiding food or smoking) and the duration of collection should be standardized to reduce pre-analytical variability and increase reproducibility [16,20]. Methodological reviews also propose uniform guidance for saliva collection, handling, and storage to ensure analytical reliability and inter-study comparability [1,11,15,22].

**Figure 2:**
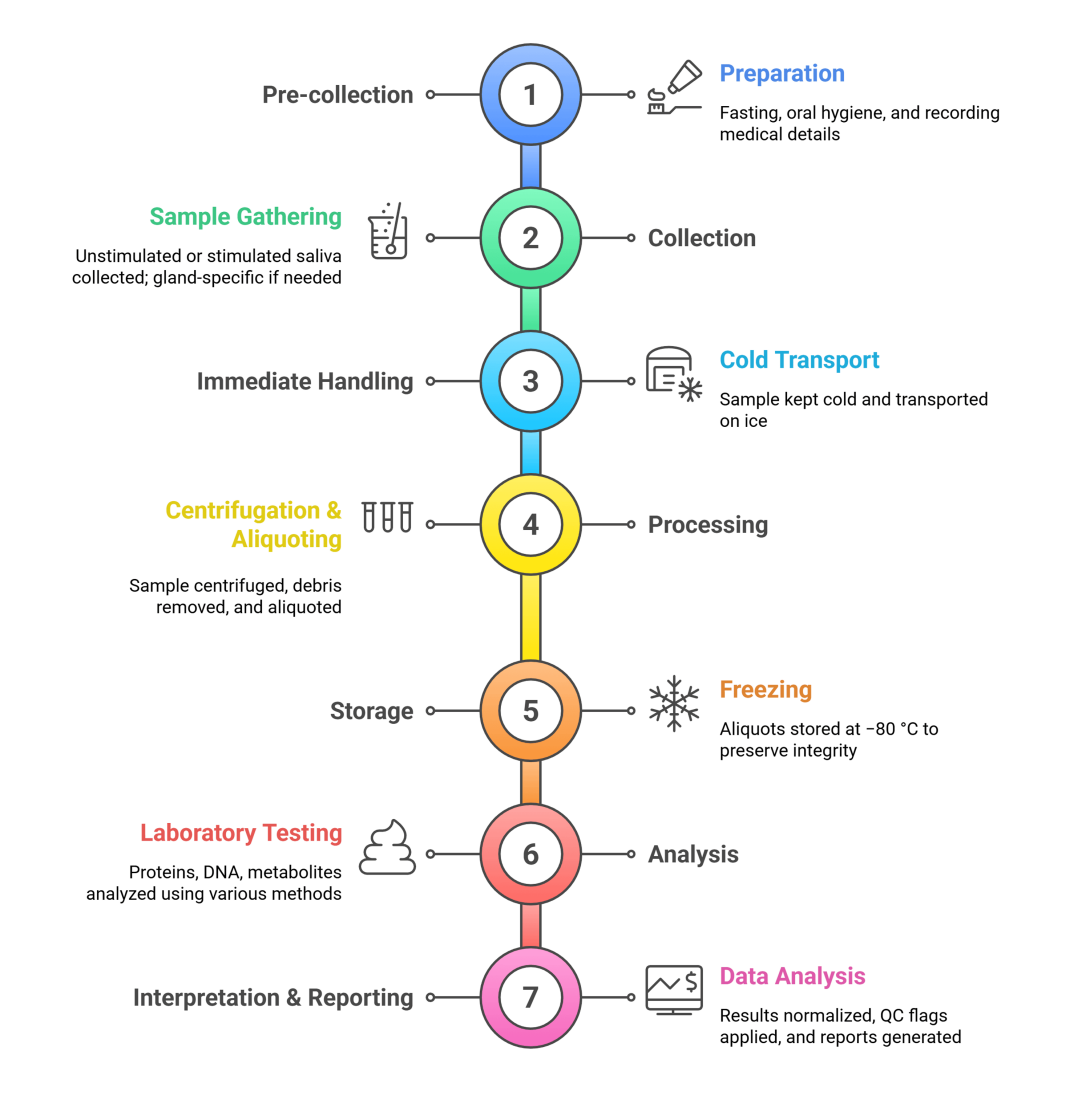
End-to-end Workflow of Salivary Diagnostics Created by authors

Prior studies comparing stimulated versus unstimulated collection, time-of-day effects, transport buffers, and pre-analytical controls provide practical guidance for pilot protocols. Based on this literature, a pilot-ready scaffold includes: (i) pre-collection fasting and oral hygiene controls; (ii) consistent time-of-day scheduling to limit circadian variability; (iii) selection of stimulated or unstimulated whole saliva aligned with the target analyte; (iv) immediate cooling, centrifugation, and aliquoting; (v) storage at −80 °C with minimized freeze-thaw cycles and protease inhibitors as needed; and (vi) predefined QC checks (e.g., visual hemolysis/gingival crevicular fluid contamination flags, duplicate blanks, spike-in recovery) [1,11,15]. Figure 2 summarizes the end-to-end steps and where each analytical method fits in the pipeline.

Processing and Storage Protocols

Salivary samples are naturally susceptible to proteolysis, bacterial contamination, and physicochemical degradation. Processing and suitable storage conditions are thus very important in maintaining the integrity of biomarkers [21]. Saliva is normally centrifuged soon after it is collected in order to get rid of cellular debris and insoluble material [14]. Supernatant fractions can be aliquoted and frozen at −80°C to store them long-term [22]. Proteins and peptides can also be stabilized by the addition of protease inhibitors [12]. In addition to this, freezing and thawing must be reduced to a minimum since the repetition of such processes may lead to the denaturation of enzymes and the degradation of nucleic acids [23]. New technology buffers and sample collection kits have enhanced the possibility of using saliva collection in the field in epidemiological studies and in point-of-care use [15,24]. The material of the container used (e.g., polypropylene tubes) as well as labeling techniques must also be considered to avoid cross-contamination and guarantee chain-of-custody during clinical research or forensic investigations [18].

Analytical Techniques

The combination of technological development over the past 20 years has greatly increased the spectrum of analytical techniques used in salivary diagnostics. The choice of method relies on the analyte of interest, the sensitivity needed, the infrastructure, and the desired clinical use [16,25]. In addition to mass-spectrometric platforms, commonly used non-spectrometric approaches include ELISA/multiplex immunoassays for proteins and cytokines (e.g., IL-6, IL-8, TNF-α), polymerase chain reaction (PCR)/qPCR and isothermal amplification (LAMP) for DNA/RNA and microRNAs, lateral-flow assays for rapid antigen/antibody detection, and electrochemical/impedance biosensors for point-of-care readouts. ELISA has been the gold standard to measure proteins, hormones, and cytokines because of its specificity, reproducibility, and scalability [11]. ELISAs are used to measure salivary interleukins, tumor necrosis factor-alpha, and immunoglobulins about oral and systemic diseases [12,19]. The detection of microbial DNA, viral RNA (e.g., SARS-CoV-2), and host nucleic acids, including microRNAs, can be attained by PCR or quantitative real-time PCR [22]. The salivary PCR assays have also been effectively used in the characterization of the pathogens causing periodontal infections and systemic viral diseases [17,23].

Mass spectrometry-based proteomics and metabolomics have emerged as a useful technique for detailed salivary composition analysis [13,20]. Protein patterns linked to diseases, including neurological illnesses and oral squamous cell carcinoma, can be promptly identified using matrix-assisted laser desorption ionization-time-of-flight (MALDI-TOF) mass spectrometry [25,26]. Liquid chromatography combined with tandem mass spectrometry (LC-MS/MS) has a greater sensitivity for post-translational alterations and low-abundance metabolites [24].

**Figure 3:**
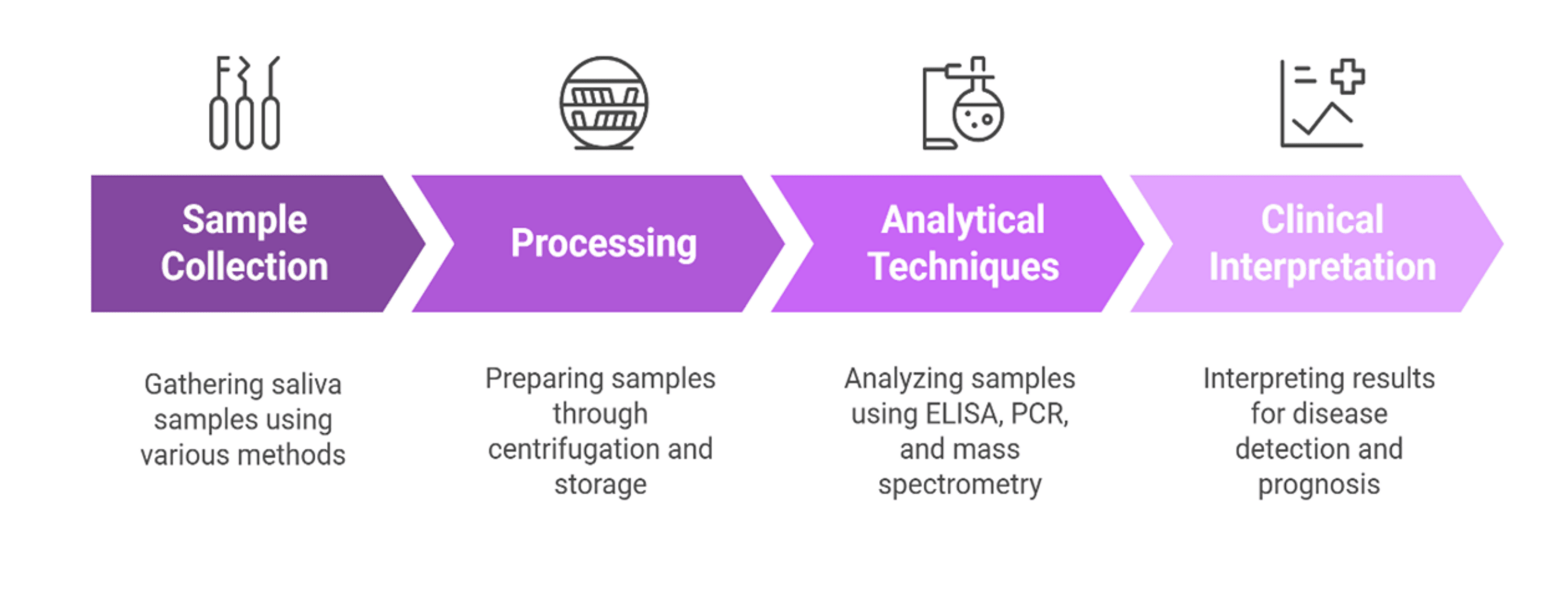
Workflow of Salivary Diagnostic Methodology Created by authors

Lastly, point-of-care saliva diagnostics is being transformed by lab-on-chip devices and biosensors. Microfluidic systems that combine immunoassays, nucleic acid amplification, and electrochemical detection are viable in terms of rapid, multiplexed analyses of biomarkers [15,27]. Such handheld devices make possible the real-time monitoring of health status and have the potential for personalized disease management and screening in resource-limited environments [28]. Figure 3 illustrates the standardized workflow for salivary diagnostics, from sample collection and processing to analytical techniques and clinical interpretation.

Salivary biomarkers in oral diseases

Dental Caries

Dental caries is among the most common oral diseases in the world, and it occurs as a result of the complex relationship between acidogenic bacteria and host-related factors, which lead to demineralization of the tooth enamel. Saliva is important in the susceptibility to caries, regulating microbial colonization, buffering ability, and mineral content [25]. Streptococcus mutans and lactobacilli are considered microbial biomarkers that are the main etiological agents. High salivary concentrations of these bacteria have also been significantly associated with caries risk, especially among individuals with poor oral hygiene and individuals who consume large amounts of sugar in their diets [26]. Other than microbiological parameters, salivary pH and buffering capacity are significant functional biomarkers of caries risk. Lowering of salivary pH below the critical level (~5.5) promotes enamel demineralization, whereas the loss of buffering capacity hinders bacterial acid neutralization [27]. Measurement of these parameters has been included in chairside risk assessment kits that may help in preventive counseling and treatment planning [28].

Periodontal Diseases

The chronic inflammatory illness known as periodontitis is linked to the breakdown of tissues that support teeth. Salivary biomarkers have shown promise in the non-invasive monitoring and detection of the activities of periodontal disease. These include the inflammatory cytokines, such as tumor necrosis factor-alpha (TNF-α) and interleukin-1 beta (IL-1β), which are invariably elevated in individuals with ongoing periodontal damage [29]. The extracellular matrix's constituents can be broken down by the proteolytic enzymes known as matrix metalloproteinases (MMPs), particularly MMP-8 and MMP-9, which have also been shown to be salivary markers of periodontal tissue degradation [30]. A new study revealed that MMP-8 salivary concentration was associated with periodontal probing depths and bleeding on probing, proving their diagnostic importance [31]. Point-of-care devices also have potential as rapid measures of salivary leukocyte esterase activity as a measure of periodontal disease severity [32]. Individually or collectively, these biomarkers can increase early diagnosis, aid in risk stratification, and oversee therapeutic response in periodontal patients [30,33].

Oral Cancer

One of the main causes of cancer-related morbidity and mortality is oral squamous cell carcinoma (OSCC). Increasing survival rates requires early diagnosis. A unique chance to see the molecular alterations that result in oral cancer is offered by saliva. Several studies have found that DNA mutations, expression of oncogenes, and epigenetic alterations in OSCC patients' salivary samples exist [26,34].

The interleukin-8 (IL-8), Cyfra 21-1, and vascular endothelial growth factor are protein markers that have shown a discriminatory level between cancerous and non-cancerous lesions [35]. Proteomics using mass spectrometry has also demonstrated tumor-stage- and prognosis-related specific salivary protein signatures [25,36]. Evidence supporting the use of salivary metabolomics in OSCC detection is mounting. Amino acids, organic acids, and polyamines salivary profiles have been demonstrated to differ significantly in patients compared to healthy controls, providing an alternative solution to protein and genetic markers [37].

Oral Potentially Malignant Disorders

Leukoplakia and erythroplakia are some of the most frequent oral potentially malignant disorders (OPMDs), with a variable risk of transformation into invasive carcinoma. In OPMDs, the salivary diagnostics aim at detecting molecular changes that precede the malignant transformation. For instance, dysplastic patients have been found to have elevated levels of cytokines, such as TNF-α and IL-6 [35].

MicroRNAs in saliva have proved to be especially attractive biomarkers in OPMDs. Dysplasia and early neoplastic changes have been linked to the overexpression of miR-21 and downregulation of tumor suppressor microRNAs in specific miRNA signature patterns [24,38]. Observation of these biomarkers might allow clinicians to introduce specific surveillance and early intervention measures and minimize the impact of late-stage cancers. Published evidence indicates that salivary cytokine, proteomic, and metabolomic signatures can distinguish OSCC and potentially malignant disorders from healthy controls and support earlier risk stratification [2,4,8]. A summary of key salivary biomarkers relevant to the diagnosis and monitoring of major oral diseases is presented in Table 1.

**Table 1:** Key Salivary Biomarkers in Oral Diseases OPMDs: Oral Potentially Malignant Disorders

Disease	Biomarker Category	Specific Biomarkers	Clinical Utility	References
Dental caries	Microbial	*Streptococcus mutans*, *Lactobacilli*	Caries risk prediction	[25–28]
Dental caries	Biochemical	Salivary pH, buffering capacity	Functional risk assessment	[27,28]
Periodontitis	Inflammatory cytokines	IL-1β, TNF-α	Disease activity monitoring	[29,30]
Periodontitis	Proteolytic enzymes	MMP-8, MMP-9	Tissue destruction indicator	[30,31]
Periodontitis	Enzyme Activity	Leukocyte esterase	Rapid severity assessment	[32,33]
Oral cancer	Proteins	IL-8, Cyfra 21-1	Early detection, prognosis	[26,34,35]
Oral cancer	Nucleic acids	DNA mutations, microRNAs (miR-21)	Tumor profiling	[24,34,38]
OPMDs	Cytokines and microRNAs	IL-6, TNF-α, miR-21	Malignant transformation risk	[24,35,38]

Salivary biomarkers in systemic diseases

Diabetes Mellitus

Diabetes mellitus is a metabolic disorder that has chronic hyperglycemia and multisystem complications. Saliva indicates systemic metabolic changes; therefore, it is a useful fluid in the management of diabetes. The patients with poorly controlled diabetes have been found to have elevated salivary glucose that correlates with the serum glucose concentrations [27,39]. Autonomic dysfunction and glycemic variability may also change the activity of salivary amylase. Moreover, diabetic patients accumulate advanced glycation end products (AGEs), which are present in saliva and are indicators of oxidative stress and chronic metabolic disproportion [28,29]. According to recent research, the inclusion of salivary biomarkers into diabetes screening guidelines may decrease the number of invasive blood tests, especially in community-based and pediatric groups of patients [30].

Cardiovascular Diseases

The number one killer in the world is cardiovascular diseases (CVDs) [40]. Salivary inflammatory and metabolic biomarkers have been explored as a possible measure of cardiovascular risk. C-reactive protein (CRP) is a well-known serum indicator of systemic inflammation that has been found in saliva and correlated with the levels in the serum and severity of disease [31,32]. There is also the investigation of salivary cardiac troponins as myocardial injury markers. Even though the sensitivity of the salivary troponin assays is still lower than that in serum, the technological advancements in the immune-sensing field can make them useful in the acute coronary syndrome [33,34]. Cardiovascular risk has also been associated with SCFAs and disrupted metabolomic profiles, which demonstrates saliva as a promising non-invasive monitoring device in high-risk individuals [25,35].

Infectious Diseases

The use of saliva in the detection of infectious diseases has been widely embraced since it contains viral particles, bacterial DNA, and immune characteristics of the host. During the COVID-19 pandemic, salivary PCR detection of SARS-CoV-2 RNA emerged as a dependable and non-invasive substitute for nasopharyngeal swabs [36]. In the same regard, salivary antibody tests have proved to be acceptable and specific in screening and monitoring HIV [37]. Salivary diagnostics in the management of infectious diseases come with logistical benefits, such as ease of collection, fewer exposure risks, and suitability to point-of-care technology [26,36].

Autoimmune Disorders

An autoimmune condition that affects the entire body, Sjögren's disease is characterized by lymphocytic infiltration of exocrine glands, which causes keratoconjunctivitis sicca in the eye and dry mouth (xerostomia). Salivary biomarkers are crucial to both the diagnosis and the tracking of this illness. Sjögren’s disease is characterized by altered salivary flow rates, altered protein composition, and increased levels of autoantibodies, such as anti-Ro/SSA and anti-La/SSB [38]. Other potential biomarkers have been found in recent proteomic studies: beta-2 microglobulin and certain cytokine profiles are other candidate biomarkers that could enhance diagnostic accuracy [25,39]. Exosomal and salivary transcriptomics are also in development as an adjunctive method to enable early diagnosis and prognosis [27,38].

Neurological Diseases

Neurodegenerative disorders, such as Parkinson’s and Alzheimer’s, can have a major impact on a person’s health. Saliva is also emerging as a possible source of biomarkers that show pathology in the central nervous system. As an example, cortisol measurements in saliva are commonly applied as markers of the hypothalamic-pituitary-adrenal axis abnormality and long-term stress, which are associated with cognitive decline [32,40]. The protein, alpha-synuclein, involved in Parkinson's disease pathogenesis, has been identified in salivary gland tissues and whole saliva. Increased concentrations of salivary alpha-synuclein have been linked to disease severity and burden of motor symptoms [25,39]. Further, a recent proteomic study showed that the salivary protein profile was able to distinguish between Alzheimer's, Parkinson's, and normal controls with good sensitivity and specificity [39]. The potential of non-invasive saliva collection as a method to monitor neurodegenerative diseases highlights its usefulness as an aid to early detection and to monitor therapy. Table 2 outlines prominent salivary biomarkers identified in systemic diseases, highlighting their diagnostic potential and supporting evidence from recent studies.

**Table 2:** Salivary Biomarkers in Systemic Diseases

Disease	Biomarker Type	Specific Biomarkers	Diagnostic Potential	References
Diabetes Mellitus	Metabolic	Glucose, amylase, advanced glycation end products (AGEs)	Glycemic status assessment	[27–30]
Cardiovascular Diseases	Inflammatory/protein	C-reactive protein (CRP), cardiac troponins	Cardiovascular risk monitoring	[31–34]
Infectious Diseases	Nucleic acids & antibodies	SARS-CoV-2 RNA, HIV antibodies	Infectious disease detection	[36,37]
Autoimmune Disorders	Autoantibodies	Anti-Ro/SSA, anti-La/SSB	Sjögren’s syndrome diagnosis	[38]
Neurological Diseases	Proteins	Alpha-synuclein	Parkinson’s disease monitoring	[25,39]
Neurological Diseases	Hormonal	Cortisol	Stress and cognitive decline	[32,40]

Technological advances in salivary diagnostics

Point-of-Care Devices

The introduction of a point-of-care (POC) device is one of the most important changes in salivary diagnostics. The platforms combine portable biosensors and microfluidic technologies to make a detection of salivary biomarkers at a rapid rate and in real time, and with minimum sample preparation. Microliter volumes of saliva can be processed in microfluidic chips, which are able to be used to perform multiplex assays to simultaneously identify proteins, nucleic acids, and metabolites [31,32].

As another example, lab-on-chip immunoassays have been used to measure inflammatory cytokines, including interleukin-6 and tumor necrosis factor-alpha, in periodontal disease and CRP in cardiovascular disease [33]. Saliva-based POC devices have proved useful in the management of infectious diseases in the context of HIV antibody and SARS-CoV-2 RNA detection in a safer and more acceptable method than invasive sampling [36].

Biosensors (both electrochemical and optical) are also being designed in a highly sensitive and specific manner, and nanomaterials like graphene oxide or gold nanoparticles are frequently used as a means of increasing the detection limits [34,37]. Oral cancer. A recent study showed that ultrasensitive microRNA detection in saliva by rolling circle amplification with a DNA-decorated graphene oxide sensor can be used to provide exemplary diagnostic performance [24,34]. These innovations are promising for decentralized screening programs, remote monitoring, and fast triage in both high-resource and low-resource environments [33].

Omics Approaches

The salivary proteome, genome, transcriptome, and metabolome profiling are being transformed by the omics technology. Proteomics in the context of mass spectrometry has discovered disease-specific protein signatures in conditions like OSCC, Sjögren syndrome, and neurodegenerative diseases [25,39].

Certain molecule patterns linked to diabetes, cardiovascular illnesses, and chronic kidney disease have been identified through the use of salivary metabolomics with the use of techniques like nuclear magnetic resonance spectroscopy and liquid chromatography-mass spectrometry [35, 40]. For example, alterations in amino acids and SCFAs have been proposed as indicators of metabolic dysregulation and systemic inflammation [25, 35]. Genomic and transcriptomic studies have also become popular, and salivary microRNAs have proved to be strong and less invasive biomarkers. Malignant and benign lesions of the oral cavity can be differentiated by specific miRNA signatures, and disease progression can be predicted [24,38]. Furthermore, salivary EVs are recently emerging as a robust delivery platform of RNA- and protein-based biomarkers, which broadens the application of saliva-based molecular diagnostics [23,41].

Integration with Digital Health

The meeting of salivary diagnostics and digital health platforms is changing the paradigm of preventive care and personalized treatment. Wearable sensors are also being made that can continuously measure the salivary parameters like pH, glucose, and electrolyte concentration in real time [37]. Such devices allow transferring the data wirelessly to smartphones or cloud-based systems, so that the disease biomarkers could be monitored continuously, and the clinical interventions could be provided in time [33,38].

Saliva-based testing is also being included in telemedicine applications as a key component of remote patient management. As an example, the virtual consultations and disease monitoring services based on saliva collection kits and smartphone-enabled biosensors will not necessitate a visit to the clinic [36,39]. This integration falls in line with the general trends of precision health, which allows patients to be more active in their treatment process and lessens the obstacles to early diagnosis and the management of chronic conditions [32,42].

**Figure 4:**
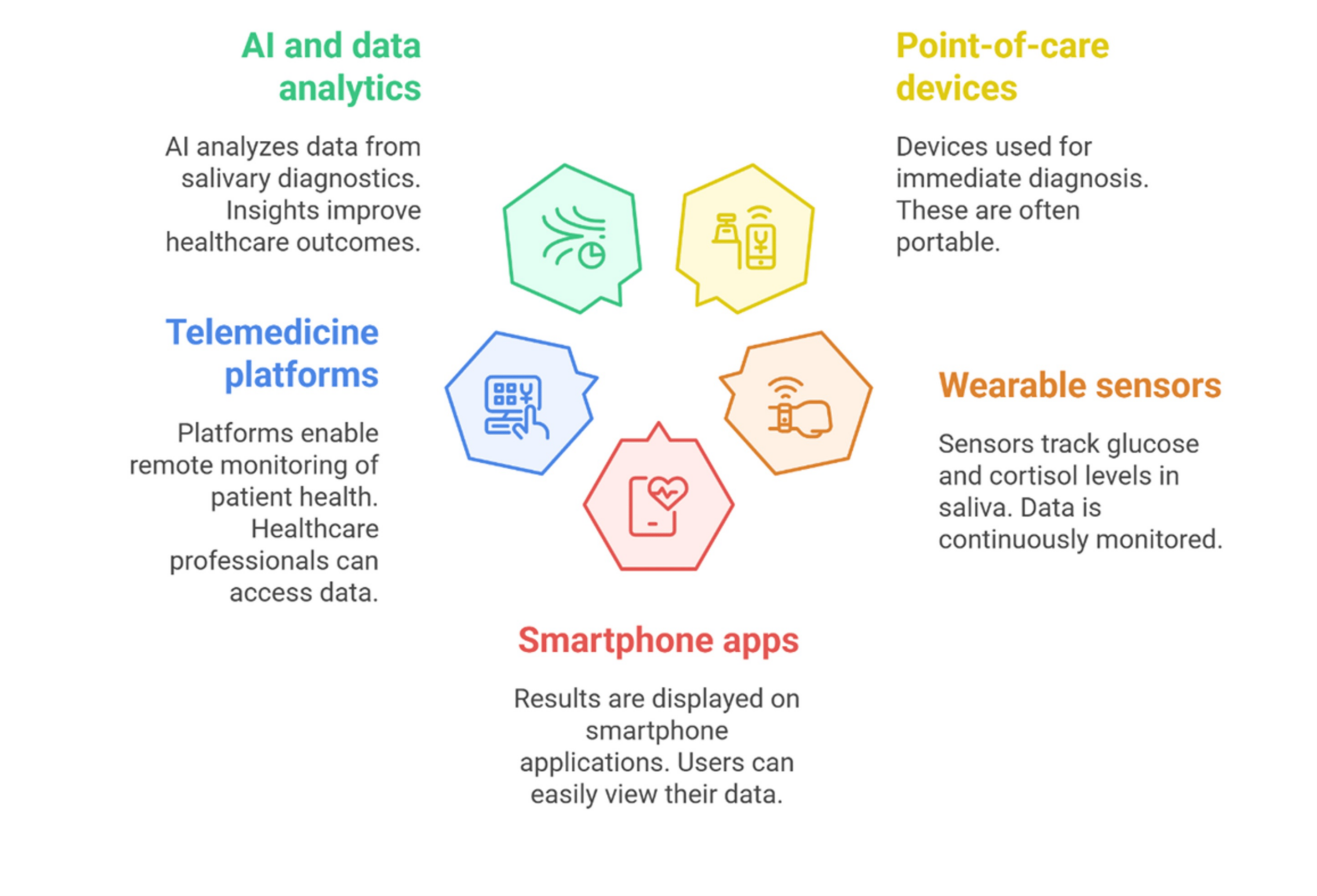
Integration of Salivary Diagnostics with Digital Health Platforms Created by authors

All these technological innovations highlight the prospects of saliva as a general-purpose diagnostic fluid. With the maturation of digital ecosystems and increasing performance of analytical systems, salivary diagnostics will become a part of modern and patient-centered healthcare. Salivary diagnostics can be seamlessly integrated with digital health solutions, including POC devices, wearable sensors, and telemedicine applications. However, the feasibility of having intraoral or mouth-based sensors for continuous salivary monitoring in normal individuals remains a challenge. Although experimental prototypes, such as mouthguard-type biosensors for glucose, pH, or electrolyte detection, have demonstrated promising accuracy and wireless data transfer, their routine clinical or daily use is still limited by issues of comfort, hygiene, long-term stability, and user compliance. Hence, while the integration of such devices with digital health platforms is technologically achievable, their real-world application in healthy or general populations will require significant ergonomic and biocompatibility refinements before widespread adoption. Figure 4 depicts the digital health ecosystem for salivary diagnostics, including POC devices, wearables, smartphone apps, telemedicine platforms, and AI analytics.

Challenges and limitations

Biological Variability

The main limitations of salivary diagnostics are the high biological variability of the salivary composition, which may interfere with the interpretation of biomarkers. This variability differs across individuals based on health conditions and habits, for example, age, sex, circadian rhythms, medications, diet, and smoking, as well as physiological factors such as stress, hydration status, and fluctuations in salivary flow, underscoring the need for rigorous, standardized sampling to mitigate inter-individual differences [43]. The genetic background, age, sex, circadian rhythms, medications, and lifestyle (diet and smoking) are the factors that generate inter-individual differences [1,14,25]. Inter-individual differences are also huge; salivary flow rates and concentrations of constituents vary throughout the day and depend on stress or dehydration [12,31]. These aspects make it difficult to obtain universal reference ranges and underscore the importance of standardized sampling procedures to accommodate such variation [16,29].

Standardization Issues

Regardless of the fast technological advancement, the best practices regarding saliva collection, processing, and storage remain undecided. The use of various collection techniques (stimulated vs. unstimulated), collection times, and analytical platforms is common in studies, and such a variety makes it hard to compare them [1,8,15]. As an example, the speed of centrifugation, addition of protease inhibitors, and storage temperature may also cause discrepancies in the stability and quantification of biomarkers [13,22,24]. These standardization concerns must be addressed to enable reproducibility as well as ease regulatory approval of salivary diagnostic tests [16,28].

Sensitivity and Specificity Concerns

Although a large number of salivary biomarkers are promising, they are not as effective as the established serum-based tests in diagnosis. Minimal analyte levels in saliva as compared to plasma may reduce sensitivity, especially when detecting early stages of the disease [2,5,26]. Moreover, blood contamination or gingival crevicular fluid can cause false-positive or false-negative results, particularly in the evaluation of inflammatory mediators or nucleic acids [3,11,19]. New microfluidics and biosensor technology have been more sensitive in analysis, although validation in large, diverse populations is needed [24,33,34].

Regulatory and Ethical Considerations

There are also regulatory and ethical issues to translate salivary diagnostics into routine practice. There are very few salivary assays that have been formally approved in clinical practice, and this is mainly because of the issue of analytical validity, clinical utility, and standardization [16,28]. Also, the privacy and data security concerns and informed consent issues arise when it comes to using genomic and proteomic data, particularly since saliva is becoming common in molecular profiling [23,38]. These issues require clear regulation and validation research, as well as assurance of clinician and patient confidence in salivary diagnostic technology [1,36].

Future perspectives

Emerging Biomarkers

Identification of novel salivary biomarkers should become a breakthrough in the field of early disease diagnosis. Exosomes and other EVs have attracted attention as relatively stable carriers of proteins, microRNAs, and DNA fragments that are indicative of systemic disease processes [23,27]. As an example, OSCC, Sjögren syndrome, and neurodegenerative diseases have been found to be differentiated using salivary exosomal microRNAs [24,38,39]. Since methods of isolation and analysis have become more standardized, likely, exosome-based assays can further improve the sensitivity and specificity of saliva-based diagnostics [23,25].

Personalized Salivary Diagnostics

The salivary testing will be personalized in the next-generation healthcare. Salivaomics’ comprehensive profiling of proteomes, transcriptomes, and metabolomes with digital health platforms will enable clinicians to individualize disease monitoring and preventive strategies for patients [7,20]. As an example, the salivary glucose or cortisol can be monitored using wearable biosensors in real-time, allowing the dynamic control of diabetes or stress-related disorders [37,40]. This direction is supported by recent reviews and methodological papers outlining AI/ML applications to saliva-omics and clinical integration [1,11,18,22,37].

Global Adoption and Accessibility

In order to achieve the potential of salivary diagnostics, approaches to its extensive implementation into primary care and public health systems are needed. Portable, inexpensive, and easy-to-use POC devices will play a pivotal role in the implementation of low-resource settings and community-based screening efforts [15,33]. Also, social education will allow raising awareness and acceptance of saliva-based testing, dispelling the myths of its effectiveness or usefulness [16,28]. Researchers, policymakers, and industry stakeholders will have to work together to standardize, achieve fair access, and develop infrastructure to scale up implementation [5,36].

## Conclusions

The physiology, composition, and clinical applications of salivary diagnostics have all been compiled in this comprehensive overview, which also highlights saliva's enormous potential as a multifunctional diagnostic fluid for systemic and oral disorders. The main value of this work is that it is an integrative analysis of the emerging salivary biomarkers, including microbial profiles and cytokines, to exosomes and microRNAs, and the technological innovations in POC devices, salivaomics, and biosensor platforms that are transforming the field of non-invasive diagnostics. Through the critical analysis of methodological issues, such as collection procedures, analytical methods, and the issue of standardization, this review will offer a guide on how the salivary diagnostics could be translated into clinical practice out of the research environment. The effect on research is significant, since it indicates how saliva can be used to detect conditions such as oral cancer, diabetes, cardiovascular diseases, and neurodegenerative disorders earlier, which positively affects prognosis, minimizes the burden of treatment, and allows implementing personalized approaches to treatment. Furthermore, the regulatory and ethics discussion highlights the need to create thorough validation schemes that will be reliable and trusted by the patients. In the future, the role of salivary diagnostics will be determined by the cooperation of different fields to identify new biomarkers, improve wearable and mobile health devices, and incorporate salivary-based tests in the everyday practice of healthcare provision in every country. Through innovation and mitigation of existing constraints, saliva can revolutionize preventive medicine and facilitate mass screening programs and democratize access to high-quality diagnostics, especially in resource-constrained environments where conventional methods will continue to be impractical or unaffordable. Moreover, population-level salivary screening has been piloted to characterize differences in disease patterns and body mass index (BMI) among minority groups, underscoring the potential of saliva-based approaches for equitable, community-focused surveillance and targeted interventions.
